# Silica–Calcite Sedimentary Rock (Opoka) Enhances the Immunological Status and Improves the Growth Rate in Broilers Exposed to Ochratoxin A in Feed

**DOI:** 10.3390/ani14010024

**Published:** 2023-12-20

**Authors:** Mateusz Makarski, Klara Piotrowska, Artur Żbikowski, Karol Pawłowski, Anna Rygało-Galewska, Maciej Szmidt, Andrzej Łozicki, Tomasz Niemiec

**Affiliations:** 1 Department of Animal Nutrition, Institute of Animal Sciences, Warsaw University of Life Sciences, 02-776 Warsaw, Poland; matmakarski@gmail.com (M.M.); anna_rygalo-galewska@sggw.edu.pl (A.R.-G.); andrzej_lozicki@sggw.edu.pl (A.Ł.); 2 Department of Pathology and Veterinary Diagnostics, Institute of Veterinary Medicine, Warsaw University of Life Sciences, 02-776 Warsaw, Poland; artur_zbikowski@sggw.edu.pl (A.Ż.); karol_pawlowski@sggw.edu.pl (K.P.); 3 Department of Morphologic Sciences, Institute of Veterinary Medicine, Warsaw University of Life Sciences, 02-776 Warsaw, Poland; maciej_szmidt@sggw.edu.pl

**Keywords:** mycotoxins, chicken, Ochratoxin A, OTA, Opoka, silica–calcite sedimentary rock

## Abstract

**Simple Summary:**

Mycotoxins, produced by certain types of mold, can present a significant challenge for farm animals, particularly chickens. Among these mycotoxins, Ochratoxin A, created by common molds, is considered one of the most harmful. The absorption of these mycotoxins by animals can be mitigated by incorporating specific substances into their feed that absorb the toxins. Opoka, a rock composed of tiny organic particles, emerges as a potential candidate for this purpose due to its unique structure, which contains numerous small spaces capable of trapping toxins. In a recent study, 2% of Opoka was added to chicken feed. The growth and immune response of the birds were measured, revealing that the weight loss induced by the mycotoxin could be counteracted by including Opoka in the feed. Chickens consuming the Opoka-enhanced feed exhibited increased white blood cells and higher blood glucose levels. These findings suggest that Opoka has the potential to play a protective role against mycotoxin-induced damage. Consequently, introducing Opoka to chicken feed appears to be a promising strategy for shielding chickens from the harmful effects of these molds.

**Abstract:**

Mycotoxins, such as Ochratoxin A (OTA), originating from fungi like Aspergillus and Penicillium, represent serious health hazards to poultry. The use of mycotoxin-adsorbing feed additives can reduce these risks. Opoka, a porous transitional rock, shows promise as one of these additives. This study is the first to examine the effect of Opoka administered with OTA on zootechnical parameters and the immune response of chickens. A 42-day investigation examined the impact of 1% of Opoka supplementation in feed on OTA-challenged broiler chickens. Seventy-two chickens were allocated into three groups of twenty-four individuals each: a control group, an OTA-exposed (2 mg/kg feed) group, and an OTA (2 mg/kg feed) plus 1% of Opoka group. Growth and blood parameters were monitored at predetermined intervals, and comprehensive biochemical, hematological, and cytometric analyses were conducted. The study showed that OTA exposure had a negative impact on chicken weight gain. However, adding Opoka to the diet improved weight gain, indicating its potential as a protective agent. Chickens fed with Opoka also had an increased white blood cell count, which suggests an improved immune response and elevated glucose and cholesterol concentrations. These findings indicate that Opoka may be useful in mitigating health complications caused by OTA exposure in broilers.

## 1. Introduction

Broilers are particularly vulnerable to harmful environmental conditions due to the combination of industrial farming conditions, high animal density, high levels of inbreeding, and rapid weight gain. Proper nutrition is a fundamental condition for the effective functioning of the immune system, which involves supplying an optimal amount of nutrients to the organism. Nutritional deficiencies can negatively impact zootechnical parameters and the development of lymphoid organs. They can also reduce the synthesis of immunologically active substances, the proliferation of lymphocytes, and their phagocytic ability. This, in turn, can lower the animals’ resistance to pathogenic factors. Poultry may require higher levels of certain essential nutrients to achieve optimal immunity and to maximize production efficiency [1,2,3].

A deficiency in total protein and energy results in decreased blood total protein, leukocytes, and T lymphocytes in the thymus. This deficiency also reduces the proliferation of splenocytes in response to phytohemagglutinin stimulation, which impairs the effectiveness of humoral immune reactions. The antibody titer after SRB antigen injection is used to quantify this impairment [4]. Feed additives can have an impact on the functioning of the immune system. For instance, a zinc deficiency can significantly reduce birds’ immunity [5]. Nutraceutical compounds play a pivotal role in modulating the normal physiological functions of animals and contribute significantly to fortifying them against infectious diseases. These compounds include antioxidants, vitamins, fatty acids, probiotics, medicinal herbs, natural extracts, and other substances [3]. Vaccinating chickens is a crucial strategy for enhancing disease resistance. A properly executed vaccination program can safeguard the flock against numerous illnesses [6].

An important issue that has yet to be resolved is the presence of mycotoxins in animal feed. Mycotoxins are common contaminants in cereal grains used for animal feed, with estimates suggesting that at least one mycotoxin affects about 25% of cereal grains worldwide, resulting in significant economic losses. Mycotoxins reduce the shelf life of cereal seeds, decrease product quality, and shorten storage duration [7].

Ochratoxin A (OTA) is a mycotoxin produced mainly by Aspergillus and Penicillium molds. OTA is a common contaminant in cereals, mainly oats and barley, as well as in animal feed derived from these grains [7,8].

During a 10-year study that analyzed feed and raw materials from 100 countries, it was found that 88% of the samples were contaminated with at least one mycotoxin. Among the animal feeds analyzed, 23% contained OTA in the 2 to 1582 µg/kg range. The majority of samples from Europe were within the limits established by the EU, while samples from Asia frequently exceeded them [9]. During a three-year study conducted in Poland, ochratoxin A was detected in 9% of feed samples. OTA residues were found in 23.5% of meat samples, but in most cases, the content did not exceed 5 μg/kg [10]. In a separate study, the average OTA content in France’s poultry meat was 0.2 μg/kg [11]. Chickens are especially susceptible to OTA toxicity. The fast growth rate of broiler chickens leads to an increased feed intake per unit of body weight, which increases their exposure to mycotoxins. Young chickens have an underdeveloped liver and an immature immune system, which may limit their ability to detoxify and eliminate mycotoxins efficiently. Broiler chickens’ susceptibility is increased by the negative effects of mycotoxins on their performance, health, and overall well-being. This can result in reduced growth rates, compromised immune function, and increased infection susceptibility [12].

The toxic effects of OTA involve direct and indirect mechanisms. The primary effects are related to OTA’s impact on the enzymes involved in phenylalanine metabolism and mitochondrial functions. A secondary mechanism induces increased lipid peroxidation in liver and kidney microsomes, stimulating NADPH-dependent and ascorbate-dependent lipid peroxidation with iron as a cofactor. The OTA–iron complex produces highly toxic hydroxyl radicals. OTA acts as a competitive inhibitor of carrier proteins on the inner mitochondrial membrane, inhibiting mitochondrial respiration. Although OTA is considered a potent teratogenic agent for chickens, it does not have the same effect on other domestic animals [8,12].

The immunosuppressive properties of OTA are well-documented. Its toxicity manifests through cytokine expression changes, a diminished antibody response, and reduced mass in organs such as the thymus, spleen, and lymph nodes [13,14].

The toxicity of ochratoxin to broiler chicks is presented in Table 1. The LD50 in chickens ranges from 2 to 4 mg/kg B.W. [15].

Birds can also be exposed to OTA through dermal absorption. According to Khan et al. (2019), subcutaneous OTA exposure reduced the size of the spleen, thymus, and bursa of Fabricius and caused leukocytopenia in broiler chicks. The study also found a decrease in circulating lymphocytes and heterophils, increased monocyte levels, and reduced serum IgY and IgA concentrations [18].

Various strategies are implemented to reduce mycotoxin prevalence in animal feed and related products to protect animals from the adverse effects of OTA ingestion [19]. It is essential to employ a variety of techniques both before planting, during plant growth, and throughout the harvesting and storage processes [19].

One key research direction involves exploring methods to reduce mycotoxin absorption in animals’ gastrointestinal system. A promising strategy is using adsorbents in feed, which bind the toxins. The existing literature suggests that incorporating clay minerals, activated carbon, modified yeast cell walls, and aluminosilicates in animal feeds afflicted with mycotoxins is an economical, relatively safe, and user-friendly method to reduce toxin bioavailability [20]. 

While there is a wealth of literature documenting various adsorbents used for protection against OTA, there are limited data on the application of Opoka. Opoka is a sedimentary rock that originated millions of years ago from single-celled algae in aquatic environments and has become a promising candidate for mycotoxin adsorption. As water bodies evaporated, the algal remnants settled and compacted into dense layers on the seabed, persisting over extended periods. These deposits are predominantly found in Eastern Europe. Opoka’s chemical composition varies regionally but typically consists of 50–70% calcium carbonate, approximately 20% nondetrital quartz, 10% opal, and 6% clays. Opoka’s porous structure gives it exceptional adsorptive properties [21,22]. 

The incorporation of Opoka in animal feed has not been extensively investigated. Montajewa et al. [23] observed that adding 3% of Opoka to quail feed sourced from Kazakhstan did not alter the meat’s proximate content; however, enhancements in the birds’ overall health were noted. Makarski et al. reported that a 1% inclusion of Opoka in broiler feed positively impacted the technological attributes of the meat [24] and increased MCHC g/dL [25].

Opoka has been used in wastewater treatments to absorb nitrogen, ammonium nitrogen, and total phosphorus [26,27]. However, it has not been commercially used in feed. Considering its high absorbency and previous studies on its safety in chickens, it appears to be a promising candidate as a mycotoxin absorbent. Opoka’s natural origin, low cost, and the possibility of modification by combustion at high temperatures are additional features that could facilitate its commercial use.

This investigation aimed to assess the effectiveness of Opoka, a natural adsorbent, in reducing the negative effects of OTA ingestion in poultry diets. The study focused on production results such as body weight and gains and blood biochemical and immunological parameters. It is worth noting that this is the first study to use rock as a mycotoxin absorbent.

The level of additives was selected based on the team’s previous research: 1% of Opoka in feed based on [24,25] and 2 mg of OTA/kg in feed based on [28].

## 2. Materials and Methods

### 2.1. Birds and Experimental Design

Seventy-two one-day-old chicks were included in the experiment. These were acquired as embryonated SPF eggs from VALO, Lohmann-Tierzucht, situated in Cuxhaven, Germany, and were incubated until hatching at the Division of Avian Diseases, Department of Pathology and Veterinary Diagnostics, Faculty of Veterinary Medicine, located in Warsaw University of Life Sciences. The one-day-old chicks were divided randomly into three groups, each with 24 birds: two experimental groups that were treated with Ochratoxin (OTA, and OTA and Opoka) and one control group (Table 2).

The birds were confined in laboratory coops and given access to standard feed and water ad libitum. All dietary compounds in the commercial chicken feed (Ekoplon, Grabki Duże, Poland) had no coccidiostats. The diets in all groups were identical, except for the inclusion of Opoka and OTA, as shown in Table 2. Additionally, the feeds were adjusted for calcium content based on the amount of CaCO_3_ present in the Opoka (169.8 g of Ca per 1 kg of rock), or the calcium value was corrected by taking into account the CaCO_3_ content of the Opoka (169.8 g of Ca per 1 kg of rock) [29].

The diets were developed in compliance with NRC requirements, and the detailed feed parameters are found in Table 3. Environmental conditions were monitored throughout the 42-day chicken-rearing experiment. The temperature gradually decreased from 32 °C to 21 °C over the initial 12 days and remained constant at 21 °C thereafter. The light/dark cycle was 23/1 h for the first seven days and 18/6 h for the remainder of the experiment, with humidity levels between 64% and 70%. Daily observations were conducted without interference from external factors. The animals’ body weight and weight gain were recorded on days 1, 14, 28, and 42.

### 2.2. Feed Additives

**Figure 1:**
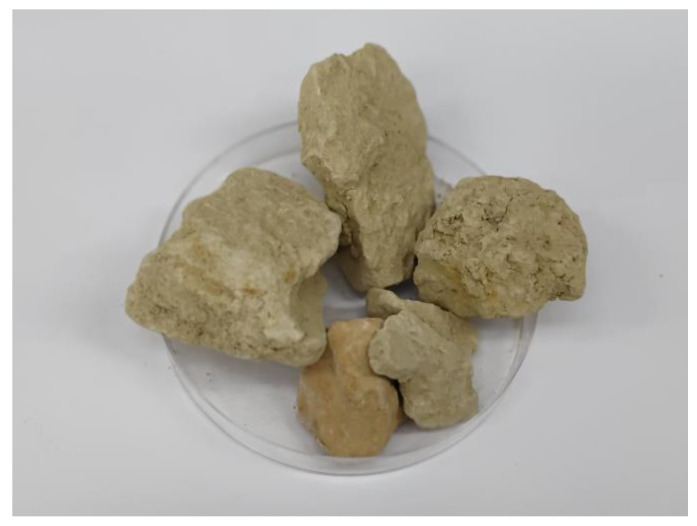
Opoka used in the experiment.

The silica–calcite sedimentary rock (Opoka) originated from deposits in the Lubelskie District, Poland (Figure 1). The raw material was cleaned and converted to micrometric particles in a ball mill (Retsch PM 100 CM). The rock mainly consists of SiO_2_ (37.2–52.1%) and CaO (19.3–28.2%). Other significant components include Al_2_O_3_ (3.8–5.6%), Fe_2_O_3_ (1.8%), and K_2_O (0.7–1.1%). Trace ingredients are present in amounts below 1%. The exact composition of the rock is detailed in the study by Brogowski and Renman [29]. Ochratoxin A (OTA) from Petromyces albertensis (≥98%, Sigma Aldrich, St. Louis, MO, USA) was used to contaminate the feed. In the OTA + Opoka group, the feed was supplemented with 1% Opoka, and in the OTA and OTA + Opoka groups, the feed was supplemented with OTA at 2 mg/kg of feed.

Feeds were adjusted for Ca content based on the CaCO_3_ contained in the Opoka (169.8 g Ca per 1 kg of rock) [29].

### 2.3. Determination of Blood Parameters

Blood samples (3 × 1 mL) were obtained from the jugular vein of six birds in each group at four different time points: 1, 14, 28, and 42 days from birth. The samples were collected into two EDTA-coated tubes for hematological and flow cytometry analyses and one tube without additives for biochemical assays. Red blood cell (RBC, 10^6^/µL) and white blood cell (WBC, 10^3^/µL) counts were quantified in a Neubauer hematological chamber using Natt and Herrick’s solution as a solvent (*n* = 6).

A chemical analyzer (Miura One, I.S.E. S.r.l., Albuccione, Italy) was used to determine the following serum biochemical parameters: aspartate transaminase (AST, U/L), alanine transaminase (ALT, U/L), alkaline phosphatase (AP, U/L), glucose (mg/dL), uric acid (mg/dL), total protein (g/L), albumin (g/L), bilirubin (mg/mL), cholesterol (mg/dL), and triglycerides (TG, mg/dL) (*n* = 6).

T and B lymphocytes and monocytes were extracted from peripheral blood using low-speed centrifugation and Histopaque^®^ 1077 (Sigma-Aldrich, St. Louis, MO, USA) density gradient separation. Specific monoclonal antibodies, including mouse anti-chicken CD3^+^ and CD4^+^ (fluorescein isothiocyanate, FITC staining), CD8^+^, Bu-1^+^ and monocytic/macrophagic^+^ antibodies (phycoerythrin, PE staining) (SouthernBiotech, Birmingham, AL, USA), were then employed. Fluorescence intensities were assessed using a BD FACSAria™ flow cytometer, and the results underwent analysis via BD FlowJo^®^ software v 10.10 (Ashland, OR, USA) (*n* = 6).

### 2.4. Statistical Analysis

The data underwent analysis via appropriate statistical tests (ANOVA, Tukey’s test) and GraphPad Prism 7.05 software (San Diego, CA, USA) and are presented as mean ± standard deviation (SD). Differences are considered significant at *p* ≤ 0.05, with a 95% acceptance rate.

## 3. Results and Discussion

This study investigated the effects of ochratoxin (OTA) and Opoka, a sedimentary rock, on broiler chicks’ growth and health parameters (Table 4). 

The group of animals receiving OTA had the lowest body weight and gains, as shown in Table 2. Animals that received additional Opoka had higher body weights and gains than those receiving OTA, and their results were comparable to the chickens in the control group. Qu et al. (2016) obtained similar results using a mixed adsorbent (sodium montmorillonite and yeast cell wall) [30], and Ghazalah et al. [31] achieved comparable results using the addition of nanosilica and bentonite. The results suggest that Opoka, similar to other tested absorbents, can prevent weight loss in broiler chickens due to mycotoxin poisoning.

During the first week of the broiler chickens’ life, the count of white blood cells (WBCs) was the lowest in both groups treated with Opoka. However, from the second week onwards, this situation began to change. In contrast, during the last week of the experiment, the highest number of WBCs was found in animals receiving Opoka (refer to Table 5). The OTA + Opoka group had more than twice as many white blood cells as the OTA group. The initial immunosuppressive effect of Opoka may be attributed to the immaturity of the birds’ immune system. It is possible that the amount of Opoka administered during this period was too high relative to the birds’ body weight and developing lymphocyte production. However, as the birds’ immune systems matured, Opoka began to have an immunostimulatory effect.

Other fossil materials have been observed to have a similar capability of increasing white blood cells in feed. As a result, diatomaceous earth has been suggested as a vaccine adjuvant for poultry [32]. Adjuvants reduce the quantity of vaccine antigens and enhance the immune response [33]. The most commonly used adjuvants in poultry vaccines are aluminum hydroxide and AS04, an oil-based adjuvant. Aluminum can lead to the production of high-titer IgG antibodies, providing prolonged immune support, ease of formulation, and relative safety. However, studies have indicated that it can accumulate in tissues, leading to hepatotoxic and nephrotoxic effects [34]. Nazmi et al. (2017) demonstrated the benefits of using DE as an adjuvant in Newcastle Disease Virus (NDV) vaccination [32]. Other studies found that it does not affect the efficacy of Infectious Bronchitis Virus (IBV) vaccines [35]. The study’s findings indicate the potential for using Opoka as an adjuvant, which warrants further research. Using natural fossil materials as adjuvants presents a more affordable alternative to current options [33]. The experimental treatments did not affect other morphological indices.

To better understand the impact of OTA and Opoka on the immune system, we examined the participation of individual types of white blood cells (Table 6). T lymphocytes are key components of the adaptive immune system. Although the values varied depending on the study week, at the end of the experiment, there was a trend that animals fed with OTA had lower lymphocyte counts in all cell types except for CD3^+^ T cells (refer to Table 4). Although these changes were not statistically significant, they are consistent with the findings of other researchers. Wang (2009) demonstrated that the administration of ochratoxin A and T-2 toxin reduced the numbers of CD4^+^ and CD8^+^ T lymphocytes in broiler chickens and also altered the typical balance between these two fractions [36]. Similarly, Ma et al. (2004) observed a reduction in lymphocytes following treatment with low doses of ochratoxin [37]. Zaleska et al. (2011) confirmed that ochratoxin A in feed reduces the activity of lymphocytes in laying hens and their offspring. This effect is attributed to the destructive impact of mycotoxins on lymphatic organs [38]. However, the application of Opoka, along with the administration of ochratoxin A, not only neutralized this trend but also increased the counts of T and B lymphocytes in relation to the control group. Recently, Lakkawar et al. (2017) demonstrated that diatomaceous earth can alleviate the pathological changes in scleral and lymphoid organs caused by mycotoxins [39]. The results of this study suggest that the use of Opoka may also support the immune system.

The health of birds in intensive systems depends heavily on the strength of their immune system, which determines the level of post-vaccination immunity and the effectiveness of treatments. The percentage of monocytes was lowest in the first week and peaked in the second and sixth weeks in the OTA and OTA + Opoka groups. Khan et al. (2019) observed a similar increase in monocytes in the blood of birds exposed to OTA during their first sampling [17]. However, the addition of Opoka significantly mitigated this effect, suggesting a potential reversal of the OTA impact. In the following weeks, temporary fluctuations were observed, but by the end of the experiment, the initial trend reappeared.

No differences in the percentage of CD45^+^ leukocytes were observed between the experimental and control groups throughout the experiment. Khan et al. (2019) discovered that the total number of leukocytes decreased in groups receiving 0.5 mg of OTA per kg of body weight and higher [17]. This discrepancy may be due to methodological differences. In the study by Khan et al., OTA was administered to chickens via subcutaneous inoculation instead of oral administration in feed, as in the current study. It is recognized that birds absorb mycotoxins from the gastrointestinal tract less efficiently than mammals [25]. This disparity could also be evident when mycotoxins are administered in feed at higher doses.

Alanine aminotransferase (ALT) and aspartate aminotransferase (AST) are enzymes released into the bloodstream from liver cells due to hepatocellular injury or changes in liver membrane permeability caused by glycogen and lipid accumulation (Table 7). These enzymes are measured for diagnostic purposes, particularly in cases of hepatitis and necrosis [40]. Other studies have shown that ochratoxin use increases broiler chickens’ AST and ALT levels [41]. The present study showed no statistically significant difference in AST levels. However, there was a slight trend towards an increase in AST levels in the groups receiving OTA and a decrease when additional Opoka was used (refer to Table 5). Awais (2022) demonstrated that supplementation with bentonite clay alone or in combination with stillage addition lowered AST values compared to the administration of OTA alone. Additionally, both study groups showed reduced OTA-induced liver lesions [41].

The increase in ALT levels during the second week of life in the group receiving OTA + Opoka, compared to those without Opoka, is concerning. This may be influenced by the aluminum content in the rock. Aluminum is known to have a hepatotoxic effect and can also increase the level of ALT in the blood of animals [34]. Furthermore, aluminum compounds, similar to Opoka, are widely used as vaccine adjuvants as they enhance the body’s immune response.

However, the changes in ALT levels after Opoka treatment were temporary. At the end of the experiment, there was a non-significant trend towards an increase in this parameter in both groups that received OTA, which is consistent with previous studies [41,42]. No effect of OTA or Opoka on alkaline phosphatase (AP), bilirubin, or triglyceride levels was observed.

In a typical course of ochratoxin poisoning, a decrease in uric acid is observed, which is associated with kidney impairment [42]. By the sixth week of the study, a clear trend was observed for the OTA-only group to have lower uric acid levels. However, the use of Opoka with OTA prevented this decrease and even tended to increase uric acid levels compared to the control group. Opoka can potentially protect kidney function, likely due to its mycotoxin-binding capacity in the feed.

In two-week-old animals, the highest cholesterol concentration was observed in those receiving OTA and OTA + OPOKA. However, from 4 weeks of age, these differences were no longer significant. By the sixth week, there was a trend towards higher cholesterol levels in the chickens receiving Opoka. Additionally, studies suggest that incorporating 1% of silicate minerals (halloysite) into chickens’ diets can lower blood cholesterol levels [43]. At the end of the experiment, there was a statistically insignificant tendency for OTA to lower cholesterol, which is consistent with previous studies [16].

During the first two weeks, both groups fed with the addition of OTA showed increased blood glucose content. As the chickens matured, these changes were more pronounced in the group receiving Opoka. This may suggest a protective effect of Opoka on the liver, which could result in more efficient glucose metabolism. 

At the beginning of the experiment, the chickens in the control group had the lowest levels of total protein and albumin. However, this difference was not significant from the second week onwards. Ochratoxicosis usually causes a decrease in total protein and albumin levels in the blood, which is associated with liver damage [43]. The absence of this outcome, particularly the opposite trend observed during the initial week, suggests that the administered dose may have been too low to cause damage to the liver cells.

## 4. Conclusions

This study examined the impact of ochratoxin (OTA) and Opoka, a sedimentary rock, on the health and development of broiler chickens. The results showed that OTA intake led to the lowest body weight and gains in broiler chickens. However, the broiler chickens that were given Opoka in addition to OTA had higher body weights and gains, indicating that Opoka may counteract OTA’s adverse effect on these parameters. This finding is consistent with previous studies using other absorbents. Opoka had a significant impact on the white blood cell count, indicating its potential to enhance the immune response of broiler chickens. These findings suggest that Opoka reduces broiler chickens’ weight loss and boosts their immune system.

In the groups receiving Opoka, both with and without OTA, the animals had an unusual increase in cholesterol, total protein, albumin, and glucose during the first two weeks. These frequent changes, which only appear in young animals, may suggest that using Opoka in the first two weeks of rearing could be detrimental. Although these changes are statistically significant, they are within the range normally observed in poultry. Nonetheless, it would be interesting to conduct further studies where the Opoka dose is adjusted according to the age of experimental animals.

## Figures and Tables

**Table 1:** Selected studies confirming the toxicity of ochratoxin A.

OTA Dose	Time	Effect	Authors
1 mg/kg B.W.	One day-old to 20-day-old chick	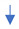 in ALP activity in the brush border 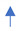 in AP activity in the cytoplasm of the epithelial cells of renal tubules.	Peckham J. et al. [15]
0.1 mg dose	One day-old chick	Dead after one day.	Peckham J. et al. [15]
0.2 mg/kg B.W.	One day-old to 6 weeks-old chick	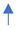 Kidney liver weight 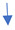 Bursa of Fabricius.	Singh et al. [16]
0.2 mg of OTA/kg in feed	One day-old to 20 weeks-old chick	No histopathological lesions indicating mycotoxin intoxication. No detectable ochratoxin residues in the kidneys, liver, and thigh and pectoral muscles.	Kozaczyński, W. [17]

**Table 2:** Description of experimental groups.

Group	Control	OTA	OTA + Opoka
OTA level in feed	0	2 mg/kg	2 mg/kg
Opoka level in feed	0	0	10 g/kg

**Table 3:** The composition of the complete dietetic commercial feed mixtures used in the experiment.

	Type of Feed		
Name	Broiler Starter Prestige, commercial diet from Ekoplon, Grabki Duże, Poland	Broiler Grower Prestige, commercial diet from Ekoplon, Grabki Duże, Poland	Broiler Finisher Prestige, commercial diet from Ekoplon, Grabki Duże, Poland
Composition	wheat, corn, post-extraction soya meal *, hemoglobin (from swine blood), rapeseed cake, soybean oil *, calcium carbonate, palm oil-derived fatty acids, monocalcium phosphate, sodium chloride	wheat, corn, post-extraction soya meal *, hemoglobin (from swine blood), rapeseed cake, post-extraction sunflower meal, palm oil-derived fatty acids, soybean oil *, swine fat, calcium carbonate, monocalcium phosphate, sodium chloride	wheat, corn, post-extraction soya meal * rapeseed cake, soybean oil *, calcium carbonate, monocalcium phosphate, sodium chloride
Component amount in 1 kg of feed			
Crude protein	225 g	205 g	187.5 g
Ether extract	48 g	55 g	75 g
Crude Fiber	27 g	30 g	33 g
Lysine	13.6 g	12.9 g	12.2 g
Methionine	6 g	5.8 g	5.6 g
Calcium	8 g	6.2 g	4.9 g
Phosphorus	6 g	5.2 g	4.6 g
Sodium	1.5 g	1.5 g	1.4 g
Ash	55 g	47 g	40 g
Dietetic additives in 1 kg of feed			
Vitamin A (3a672a)	10,000 IU		
Vitamin D3 (E671)	5000 IU		
Vitamin E (dl-α-tocopherol)	75 mg		
Fe (iron sulphate, E1)	40 mg		
J (potassium iodide, 3b201)	1.25 mg		
Cu (copper sulphate, E4)	16 mg		
Mn (manganese oxide, E5)	120 mg		
Zn (zinc oxide, E6)	100 mg		
Se (sodium selenite, E8)	0.3 mg		
Zootechnics additives in 1 kg of feed			
6-phytase (EC 3.1.3.26) 500 FTU/g, 4a19	1000 FTU	1000 FTU	-
6-phytase (EC 3.1.26) 2500 OTU/g, 4a16	-	-	250 OTU/g
Endo-1,4-β-xylanase (EC 3.2.1.8), 12,500 VU/mL, 4a22	1250 VU	1250 VU	-
Endo-1,4-β-xylanase (EC 3.2.1.8), 30,000 EPU/g, 4a1617	-	-	1500 EPU
Endo-1,3 (4)-β-gluconate (EC 3.2.1.6), 8600 VU/mL, 4a22	860 VU	860 VU	-
Serine protease (EC 3.4.21), 75,000 PROT/g 4a13	15,000 PROT	-	-

EPU—one endo-1,4-β-xylanase unit is the amount of enzyme which releases 0.0083 μmol of reducing sugars (xylose equivalent) per minute from oat spelt xylan at pH 4.7 and 50 °C; FTU—one 6-phytase unit is the amount of enzyme which releases 1 µmol of inorganic phosphate from sodium phytate in one minute at 37 °C and pH 5.5; PROT—one protease unit is the amount of enzyme which releases 1 µmol of p-nitroaniline from 1 mM substrate (Suc-Ala-AlaPro-Phe-pNA) in one minute at pH 9.0 and temperature 37 °C; VU—one endo-1,3(4)-β-gluconate unit is the amount of enzyme which hydrolyses substrate (β-glucan of barley and arabinoxylan of wheat, respectively), while reducing viscosity of the solution, so that there is a change in relative fluidity of 1 (dimensionless unit) in one minute at 30 °C and pH 5.5; *—produced from GMO soybeans (MON 40-3-2).

**Table 4:** Body weights and gains of broiler chickens from the group receiving control feed, feed with ochratoxin and Opoka, and feed with only ochratoxin. Different letters indicate statistically significant group differences (*p* < 0.05).

Parameter (g)	Week	Control	OTA		SE Pooled	*p*-Value
			-	Opoka		
Body weight	1	102.0 a	91.33 b	89.3 b	1.65554	0.0001
	2	190.7	152.9	173.2	14.5009	0.2164
	4	378.2	367.8	368.2	17.8893	0.8985
	6	587.3 a	485.3 b	539.4 ab	21.6097	0.0155
Gains	2	88.7	78.2	83.8	7.91185	0.6543
	4	187.5	198.3	195.0	21.0496	0.9337
	6	209.2 a	117.5 b	177.3 a	19.3744	0.0138

**Table 5:** Blood parameters of broiler chickens fed with control feed, feed with ochratoxin, and feed with ochratoxin and Opoka. Different letters indicate statistically significant group differences (*p* < 0.05).

Parameter	Week	Control	OTA Alone Opoka + OTA		SE Pooled	*p*-Value
RBC ×10^12^/L	1	2.58	1.77	2.15	0.2601	0.1266
	2	2.49	3.63	2.19	0.1701	0.0826
	4	2.64	3.18	2.27	0.3119	0.1554
	6	2.74	2.79	3.90	0.4068	0.1054
WBC ×10^9/^L	1	15.44 a	11.25 b	10.56 b	1.2530	0.0305
	2	20.06	12.72	20.20	2.6287	0.1029
	4	13.95	13.45	13.82	2.0682	0.9848
	6	16.05 a	12.50 a	26.76 b	2.8745	0.0084
OB. (mm)	1	2.14	3.03	2.32	0.3676	0.2682
	2	2.33	3.16	2.02	0.4493	0.2014
	4	3.83	3.33	3.16	0.5052	0.6333
	6	2.83	3.01	2.83	0.7864	0.9852

**Table 6:** Lymphocyte, leukocyte, and monocyte levels in the blood of broiler chickens fed with control feed with ochratoxin and feed with ochratoxin and Opoka. Different letters signify statistically significant group differences (*p* < 0.05).

Parameter	Weeks	Control	OTA Alone Opoka + OTA		SE Pooled	*p*-Value
Lymphocytes CD4 (%)	1	22.2	19.4	9.9	3.8448	0.0763
	2	7.4 a	5.6 a	12.6 b	1.1301	0.0032
	4	12.5	13.5	11.2	0.9759	0.2690
	6	9.8	8.6	11.5	0.8573	0.0973
Lymphocytes CD8 (%)	1	11.55	9.75	7.32	3.336	0.7002
	2	7.5	8.7	8.5	2.6581	0.9495
	4	11.1 b	10.7 b	20.6 a	2.3852	0.0153
	6	15.5	9.3	17.5	3.0701	0.1778
Lymphocytes CD4/8 (%)	1	3.6 b	8.2 a	3.8 b	0.74811	0.0007
	2	4.9	4.0	3.9	1.9185	0.9192
	4	7.2 ab	6.1 b	12.8 a	1.9133	0.0557
	6	5.2	2.9	4.5	1.576	0.5946
Lymphocytes T (CD3) (%)	1	29.7 a	12.3 b	8.3 b	2.7608	0.0001
	2	4.4 b	6.1 ab	9.3 a	1.2553	0.0431
	4	6.1	7.2	5.5	0.5032	0.0886
	6	4.3 b	4.7 b	7.7 a	0.606	0.0024
Lymphocytes B (%)	1	13.5	8.7	1.4	2.2050	0.0052
	2	1.2	1.4	1.9	0.2375	0.1297
	4	3.8 b	3.2 b	5.6 a	0.4442	0.0048
	6	2.9	2.7	3.7	0.5461	0.4331
Leukocytes CD45 (%)	1	97.0	94.8	98.4	1.61208	0.3044
	2	98.1	89.4	97.7	4.0374	0.2634
	4	98.7	98.6	98.8	0.3877	0.9007
	6	98.2	96.8	96.9	0.4631	0.0897
Monocytes (%)	1	11.8 b	12.7 b	1.3 a	2.2232	0.0040
	2	1.0 b	1.1 b	2.0 a	0.2197	0.0119
	4	3.8	3.8	4.1	0.2275	0.5207
	6	1.2	1.5	2.0	0.2071	0.0528

**Table 7:** Blood biochemistry parameters of broiler chickens fed with control feed, feed with ochratoxin, and feed with ochratoxin and Opoka. Different letters signify statistically significant differences between groups (*p* < 0.05).

Parameter	Week	Control	OTA		SE Pooled	*p*-Value
			-	Opoka		
AST (U/L)	1	124.3	134.8	110.7	16.079	0.5806
	2	125.0	133.1	127.7	13.628	0.9127
	4	175.9	172.3	171.3	2.9652	0.5336
	6	169.0	176.3	173.6	2.5060	0.1517
ALT (U/L)	1	7.6	9.8	10.5	1.3942	0.3000
	2	5.1 b	6.9 b	14.1 a	1.4926	0.0017
	4	6.9	8.1	8.9	2.5218	0.8575
	6	7.8	10.5	10.2	1.7005	0.4929
AP (U/L)	1	1510.4	2274.5	1825.5	284.562	0.1959
	2	1857.7	1315.5	2107.4	287.64	0.1724
	4	824.4	881.1	1027.5	139.947	0.5822
	6	737.83	843.08	778.60	51.1920	0.3663
Bilirubin (mg/dL)	1	0.6	0.6	1.3	0.3146	0.2463
	2	0.6	0.4	0.8	0.1605	0.3606
	4	0.4	0.4	0.5	0.0623	0.6445
	6	0.6	0.4	0.4	0.0701	0.4182
TG (mg/dL)	1	52.2	70.6	62.2	6.36180	0.1566
	2	81.4	73.0	68.8	5.83738	0.3257
	4	48.9	43.2	58.5	4.79712	0.1089
	6	36.9	41.5	49.9	4.09480	0.1071
Cholesterol (mg/dL)	1	114.1 a	192.2 b	171.5 b	7.94	0.0001
	2	145.5 b	178.4 a	178.9 a	7.5376	0.0095
	4	152.1	163.5	179.1	6.942	0.0457
	6	162.3	147.1	164.5	9.25960	0.3750
Glucose (mg/dL)	1	179.5 a	213.1 b	234.2 b	7.1257	0.0003
	2	268.8 a	231.2 b	245.3 b	5.3891	0.0007
	4	225.5 a	240.6 b	257.2 c	6.8204	0.0173
	6	226.8 a	224.5 a	248.4 b	4.9354	0.0071
Uric acid (mg/dL)	1	6.3	16.3	8.3	3.7071	0.1681
	2	11.8	10.1	13.7	2.8210	0.6677
	4	2.8	4.7	5.0	0.94691	0.2383
	6	4.0	2.8	5.2	0.6685	0.0752
Total Protein (g/L)	1	19 a	27.5 b	24.8 b	1.5068	0.0037
	2	26.7	29.2	28.2	1.1952	0.3473
	4	27.3	26.5	28.8	0.78646	0.1387
	6	26.3	26.6	28.5	0.66801	0.0774
Albumin (g/L)	1	11 a	16.3 b	13.8 c	0.6339	0.0001
	2	14.8	15.7	16.2	0.5494	0.2513
	4	14.2 a	15.6 b	16.8 b	0.4594	0.0035
	6	14.16	14.50	14.50	0.4194	0.8125

## Data Availability

The raw data presented in this paper are available from the authors on request.
